# The Rapid-Heat LAMPellet Method: A Potential Diagnostic Method for Human Urogenital Schistosomiasis

**DOI:** 10.1371/journal.pntd.0003963

**Published:** 2015-07-31

**Authors:** Javier Gandasegui, Pedro Fernández-Soto, Cristina Carranza-Rodríguez, José Luis Pérez-Arellano, Belén Vicente, Julio López-Abán, Antonio Muro

**Affiliations:** 1 IBSAL-CIETUS (Instituto de Investigación Biomédica de Salamanca-Centro de Investigación de Enfermedades Tropicales de la Universidad de Salamanca), Facultad de Farmacia, Universidad de Salamanca, Salamanca, Spain; 2 Departamento de Ciencias Médicas y Quirúrgicas, Facultad de Ciencias de la Salud, Universidad de Las Palmas de Gran Canaria, Las Palmas de Gran Canaria, Spain; George Washington University, UNITED STATES

## Abstract

**Background:**

Urogenital schistosomiasis due to *Schistosoma haematobium* is a serious underestimated public health problem affecting 112 million people - particularly in sub-Saharan Africa. Microscopic examination of urine samples to detect parasite eggs still remains as definitive diagnosis. This work was focussed on developing a novel loop-mediated isothermal amplification (LAMP) assay for detection of *S*. *haematobium* DNA in human urine samples as a high-throughput, simple, accurate and affordable diagnostic tool to use in diagnosis of urogenital schistosomiasis.

**Methodology/Principal Findings:**

A LAMP assay targeting a species specific sequence of *S*. *haematobium* ribosomal intergenic spacer was designed. The effectiveness of our LAMP was assessed in a number of patients´ urine samples with microscopy confirmed *S*. *haematobium* infection. For potentially large-scale application in field conditions, different DNA extraction methods, including a commercial kit, a modified NaOH extraction method and a rapid heating method were tested using small volumes of urine fractions (whole urine, supernatants and pellets). The heating of pellets from clinical samples was the most efficient method to obtain good-quality DNA detectable by LAMP. The detection limit of our LAMP was 1 fg/µL of *S*. *haematobium* DNA in urine samples. When testing all patients´ urine samples included in our study, diagnostic parameters for sensitivity and specificity were calculated for LAMP assay, 100% sensitivity (95% CI: 81.32%-100%) and 86.67% specificity (95% CI: 75.40%-94.05%), and also for microscopy detection of eggs in urine samples, 69.23% sensitivity (95% CI: 48.21% -85.63%) and 100% specificity (95% CI: 93.08%-100%).

**Conclusions/Significance:**

We have developed and evaluated, for the first time, a LAMP assay for detection of *S*. *haematobium* DNA in heated pellets from patients´ urine samples using no complicated requirement procedure for DNA extraction. The procedure has been named the Rapid-Heat LAMPellet method and has the potential to be developed further as a field diagnostic tool for use in urogenital schistosomiasis-endemic areas.

## Author Summary

Human schistosomiasis is a disease caused by several species of parasitic worms of the genus *Schistosoma* that is affecting 200 million people, especially in sub-Saharan Africa. Most people are infected with *Schistosoma haematobium*, the species that causes urogenital schistosomiasis and also bladder cancer in many chronic infections. The definitive diagnostic test is based on microscopic examination of urine samples to detect parasite eggs. This method has low sensitivity, high day-to-day variability and cannot be carried out in the acute phase of the disease since the parasite has not started yet to lay eggs. New high-throughput diagnostic tools would be desirable, permitting early treatment and preventing the pathology associated with chronic infections. An interesting approach is the loop-mediated isothermal amplification (LAMP) technique because of its simplicity in operation and potential use in clinical diagnosis and surveillance of infectious diseases. In this study, we developed and evaluated a LAMP assay for detection of *S*. *haematobium* DNA in patients´ urine samples using heated pellets with no complicated requirement procedure for DNA extraction, namely the Rapid-Heat LAMPellet method. This is a new, easy, rapid and cost-effective LAMP method that should prove useful for mass screening in limited-resource settings in urogenital schistosomiasis-endemic areas.

## Introduction

Human schistosomiasis, a parasitic freshwater snail transmitted disease caused by several species of genus *Schistosoma* trematode worms, is one of the 17 neglected tropical diseases (NTDs) considered by World Health Organization (WHO) [1]. It is estimated that 732 million persons are at risk of infection worldwide and over 200 million people are infected with this disease in 74 different countries, especially in sub-Saharan Africa [2–4], where both associated morbidity and mortality are a significant barrier to social and economic development [5–7]. It must be also observed that the prevalence of imported schistosomiasis is increasingly a problem in non-endemic areas due to the growing number of international travellers to endemic areas, expatriates and immigrants from endemic countries [8–10]. Although humans are mainly infected by five species of schistosomes, namely *Schistosoma mansoni*, *S*. *haematobium*, *S*. *japonicum*, *S*. *mekongi*, and *S*. *intercalatum*, the main burden of disease in sub-Saharan Africa is usually attributed to two species referred to as the major human schistosomes: *S*. *mansoni*, causing hepatic and intestinal schistosomiasis and *S*. *haematobium*, the chief cause of urogenital schistosomiasis [3].

More people are infected with *S*. *haematobium* than with the other schistosomes; it is estimated that 112 million people suffer from urogenital schistosomiasis [11–14]. The infection typically results in haematuria, anaemia, dysuria and genital and urinary tract lesions, but in severe cases it may also lead to kidney damage. It is well known that the deposition of *S*. *haematobium* eggs eventually leds to squamous cell carcinoma of the bladder in many chronically infected individuals [15, 16] the International Agency for Cancer Research (IACR) in association with WHO classified *S*. *haematobium* as a Group 1 biological carcinogen [17]. Moreover, most of women infected with *S*. *haematobium* suffer from female genital schistosomiasis of the lower genital tract [13]; which impairs fertility [18] and also increases susceptibility of the woman to HIV [19].

For the diagnosis of urogenital schistosomiasis, the *gold standard* remains microscopic detection of excreted ova in urine samples [20] after using either sedimentation/centrifugation or filtration methods [21]. These conventional methods are inexpensive, easy to perform under field conditions and relatively rapid. However, parasitological diagnosis has classically low sensitivity, especially in low-grade infections and may be affected by day-to-day variability in egg excretion, often missing diagnosis by microscopy [22, 23]. In addition, egg count-based criteria cannot be carried out in the acute phase of the disease since the parasite have not yet started to produce eggs. The collection of a larger number of urine samples per individual on consecutive days instead of a single one may increase the sensitivity of microscopic detection, but is more expensive and also time-consuming [23]. Identifying blood in the urine-micro or macrohaematuria- has been widely and successfully used as a good indicator of *S*. *haematobium* infection, mainly in a high prevalence situation. However, haematuria is a nonspecific symptom of urogenital schistosomiasis in areas of low endemicity and can be incorrectly estimated depending on the infection prevalence in an area [24, 25]. Antibody-based assays are useful to confirm *S*. *haematobium* infections, but do not distinguish active infection from past exposure, and so low sensitivity and specificity results frequently occur. Moreover, antibody tests are usually negative during acute symptomatic urogenital schistosomiasis. On the other hand, assays that detect circulating antigens seem very promising in the early phase of infection but still lack sensitivity in the diagnosis of light infections [20, 26, 27].

To overcome the drawbacks of both classical parasitological and immunological diagnostic methods, the development of new, more sensitive and specific molecular diagnostic tools for the diagnosis of urogenital schistosomiasis are desirable and still needed. In recent years, several studies have reported the utility of polymerase chain reaction (PCR)-based assays for sensitive and specific detection of *S*. *haematobium* DNA in human urine [28–30] and serum [31] samples. However, the PCR-based technologies are not widely used in low-income *S*. *haematobium* endemic countries because skilled operators and costly equipment are needed.

In this way, the loop-mediated isothermal amplification (LAMP) assay [32] offers a field-friendly alternative to PCR-based technologies as it is less time consuming than PCR and can be performed using a simple heating block or water bath, with results read by the naked eye under natural or UV light [33, 34]. Additionally, LAMP reagents can be storage at room temperature for weeks [35], the reaction shows low susceptibility to typical inhibitory compounds occurring in samples [36–38], its robustness against variation of reaction conditions such as pH and temperature has been described [39] and it can operate with minimal handling and processing of DNA samples for amplification [40], [41–43], or even without prior DNA extraction [36]. Thereby, considering these salient advantages over most DNA-based amplification tests, LAMP technology shows a potential use in clinical diagnosis and surveillance of infectious diseases, particularly under field conditions for most NTDs [44, 45].

Several successful approaches for LAMP assay for *Schistosoma* spp. detection have been recently reported in laboratory settings using experimentally infected animals, such as *S*. *japonicum* in rabbits [46, 47] or *S*. *mansoni* in mice [48], as well as in field settings for monitoring infected snails with *S*. *mansoni*, *S*. *haematobium* [49, 50] and *S*. *japonicum* [51, 52]. Additionally, a LAMP to detect *S*. *japonicum* in human sera has been also reported [53].

Thus, with the aim to develop new, applicable and cost-effective molecular tools for the diagnosis of urogenital schistosomiasis, in our work we have developed a new sensitive and specific LAMP assay for detection of *S*. *haematobium* in human urine samples. In this study, the effectiveness of the LAMP assay was evaluated in a number of patients´ urine samples with parasitological proven infection with *S*. *haematobium*. Different fractions of urine samples (whole urine, supernatants and pellets) as well as different methods for DNA extraction were used to compare results and cost-effectiveness. To the best of our knowledge, this is the first report using LAMP assay for detection of *S*. *haematobium* in human urine samples.

## Methods

### Ethics statement

Human urine samples used in this study were obtained as part of public health activities at Hospital Universitario Insular, Las Palmas de Gran Canaria, Spain. Later, samples were sent and stored at CIETUS, University of Salamanca, Spain, for further analyses. Human urine samples were not collected specifically for this study and all were obtained under written informed consent and coded and tested as anonymous samples. Participation of healthy urine donors for obtaining simulated artificial urine samples was voluntary. All participants were given detailed explanations about the aims, procedures and possible benefit of the study. The study protocol was approved by the institutional research commission of the University of Salamanca. Ethical approval was obtained from the Ethics Committee of the University of Salamanca (protocol approval no. 48531).

### Urine samples collection

#### Patients´ urine samples

A total of 94 human urine samples were selected from a set of samples collected from patients attending during May 2002 to April 2009 at Hospital Universitario Insular, Las Palmas de Gran Canaria, Spain, as part of public health diagnostic activities. All these patients were suggested to several parasitological diagnostic tests for suspected infectious diseases by specialized technicians according to standard routine laboratory procedures. Among the 94 human urine samples selected, a number of 78 were obtained from Sub-Saharan immigrants with a microscopy-confirmed infection of 39 samples with: *S*. *haematobium* (n = 18), *S*. *mansoni* (n = 7), several helminths (n = 9)-counting four infections with hookworms, two with *Strongyloides stercoralis*, two with *Trichuris trichiura*, one with *Enterobius vermicularis* and one mixed infection with *Loa loa*, *Mansonella perstans* and *T*. *trihiura*-, and other infectious agents (n = 5)-including protozoa (*Plasmodium falciparum*, *Giardia duodenalis*, *Trichomonas vaginalis)*, bacteria *(Chlamidia trachomatis)* and virus (hepatitis B virus; HBV). A set of urine samples from patients with eosinophilia without a confirmed diagnosis (n = 15) as well as a set of urine samples from patients without either eosinophilia and no apparent disease (n = 24) were also selected. Additionally, urine samples from healthy non-endemic individuals (n = 16) were included to use in the study as negative controls samples. All patients´ urine samples were collected using sterile plastic containers and a volume of approximately 10 mL each was stored at -80°C without the addition of any preservative or chemical until sending to CIETUS, Salamanca, Spain, for further molecular analyses.

#### Artificial urine samples

Fresh urine was collected from healthy staff donors with no history of travel to endemic area of schistosomiasis. The collected urine was divided into aliquots of 100 μL each and then artificially spiked with 2 μL of 10-fold serially diluted *S*. *haematobium* DNA ranging from 50 ng/μL to 0.5 atg/μL, thus resulting in a set of simulated urine samples with a final parasite DNA concentration ranging from 1 ng/μL to 0.01 atg/μL. Once prepared, these urine samples were stored at -20°C until further use for DNA extraction and to test the sensitivity of the LAMP assay.

### DNA extraction optimization

#### Parasites DNA samples

Genomic DNA from adult male and female *S*. *haematobium*, Egiptian Strain, NR-31682, was obtained from the Schistosomiasis Resource Center for distribution by BEI Resources, NIAID, NIH (https://www.beiresources.org/Collection/51/Schistosome-Resource-Centers.aspx). The original supplied *S*. *haematobium* DNA concentration (100 ng/μL) was confirmed by measuring in a Nanodrop ND-100 spectrophotometer (Nanodrop Technologies) and then was diluted with ultrapure water to a final concentration of 50 ng/μL. Subsequently, serial 10-fold dilutions were also prepared with ultrapure water ranging from 5 ng/μL to 0.5 atg/μL and stored at -20°C until used. DNA thus prepared was used as positive control in all PCR and LAMP reactions and also to assess sensitivity of both assays. In addition, *S*. *haematobium* DNA was used to prepare the artificial urine samples as mentioned above.

To determine the specificity of both PCR and LAMP assays, a panel of 20 DNA samples from several other helminths and protozoa were used as heterogeneous control samples including, *S*. *mansoni*, *S*. *japonicum*, *S*. *bovis*, *Fasciola hepatica*, *Loa loa*, *Brugia pahangi*, *Strongyloides venezuelensis*, *Dicrocoelium dendriticum*, *Calicophoron daubneyi*, *Hymenolepis diminuta*, *Taenia taeniformis*, *Anisakis simplex*, *Trichinella spiralis*, *Echinococcus granulosus*, *Cryptosporidium parvum*, *Giardia intestinalis*, *Entamoeba histolytica*, *Plasmodium vivax*, *P*. *ovale* and *P*. *malariae*. Concentration of these DNA samples was also measured by using a Nanodrop ND-100 and then also diluted with ultrapure water to a final concentration of 0.5 ng/μL. All these DNA samples were kept at -20°C until use in molecular assays.

#### Patients´ urine samples processing

Three different methods for DNA extraction were evaluated from eighteen patients´ urine samples with parasitologically confirmed *S*. *haematobium* infections, to obtain DNA to be used as a template in later LAMP amplification: a commercially available DNA extraction kit, a modified hot sodium hydroxide (NaOH) extraction method and a rapid heating urine sample method. Additionally, these methods were tested with different sets of aliquots obtained from each urine sample to compare results, including whole urine, urine supernatant and urinary sediment (pellets). These sets of aliquots were prepared as follows. After thawing, three whole urine aliquots of 100 μL, as well as three whole urine aliquots of 2 mL were taken from each urine sample in new clean 2 mL microcentrifuge tubes. Later, aliquots of 2 mL were centrifuged at 5000 rpm for 5 min at room temperature (RT) to pellet the urinary sediment (insoluble fraction of urine) and maintain the supernatant (soluble fraction of urine). Then, a volume of 100 μL was recovered from the supernatant and transferred to a new clean test tube. Excess supernatant was discarded but maintaining a minimal volume of 100 μL to resuspend the urinary sediment (pellet) at the bottom of the tube. In this way, from each patient´s urine sample we finally obtained three aliquots of 100 μL containing whole urine, three aliquots of 100 μL containing supernatant and three aliquots of 100 μL containing resuspended pellet. Afterwards, each type of these aliquots-whole, supernatant and pellet- was used to obtain DNA by using the three different extraction methods assayed.

### DNA extraction

In the first procedure for DNA extraction we used the i-genomic Urine DNA Extraction Mini Kit (Intron Biotechnology, UK) following the manufacturers´ instructions. DNA samples thus obtained were stored at -20°C until use in LAMP reactions.

In the second procedure, we used the hot NaOH extraction method [54] with minimal modifications in the standard protocol by adding sodium docecyl sulfate (SDS) to ensure disruption of the *S*. *haematobium* eggs to release the DNA. Briefly, an equal volume of a 50 mM NaOH solution containing 0.1% of SDS was added to urine aliquots of 100 μl and then heated at 95°C for 30 min. Subsequently, the tubes were centrifugated at 5000 rpm for 5 min and a volume of 50 μL of supernatant was recovered in a new clean tube and mixed with an equal volume of a 1 M Tris-HCl solution at pH 8.0. Each new solution thus obtained was stored at -20°C until further use as template in LAMP assays.

In the third procedure,-named the “Rapid-Heat LAMP method”*-*, each aliquot of whole urine, supernatant and pellet obtained from each urine sample was heated at 95°C for 15–20 min and then briefly spun to pellet the debris. After this, 2 μL of the supernatant were used immediately as template for each LAMP reaction. The remaining volume of each sample was stored at -20°C. To obtain DNA to be used as template in LAMP reactions to test the remaining 76 clinical urine samples included in the study, we firstly obtained the urinary sediment (pellet) as already indicated and, subsequently, the Rapid-Heat LAMP method was applied.

#### Artificial urine samples DNA obtaining

DNA was extracted from simulated artificial urine samples by using both the i-genomic Urine DNA Extraction Mini Kit (Intron Biotechnology, UK) following manufacturers´ instructions and the in house procedure, the Rapid-Heat LAMP method.

### *S*. *haematobium* LAMP primer design

**Fig 1:**
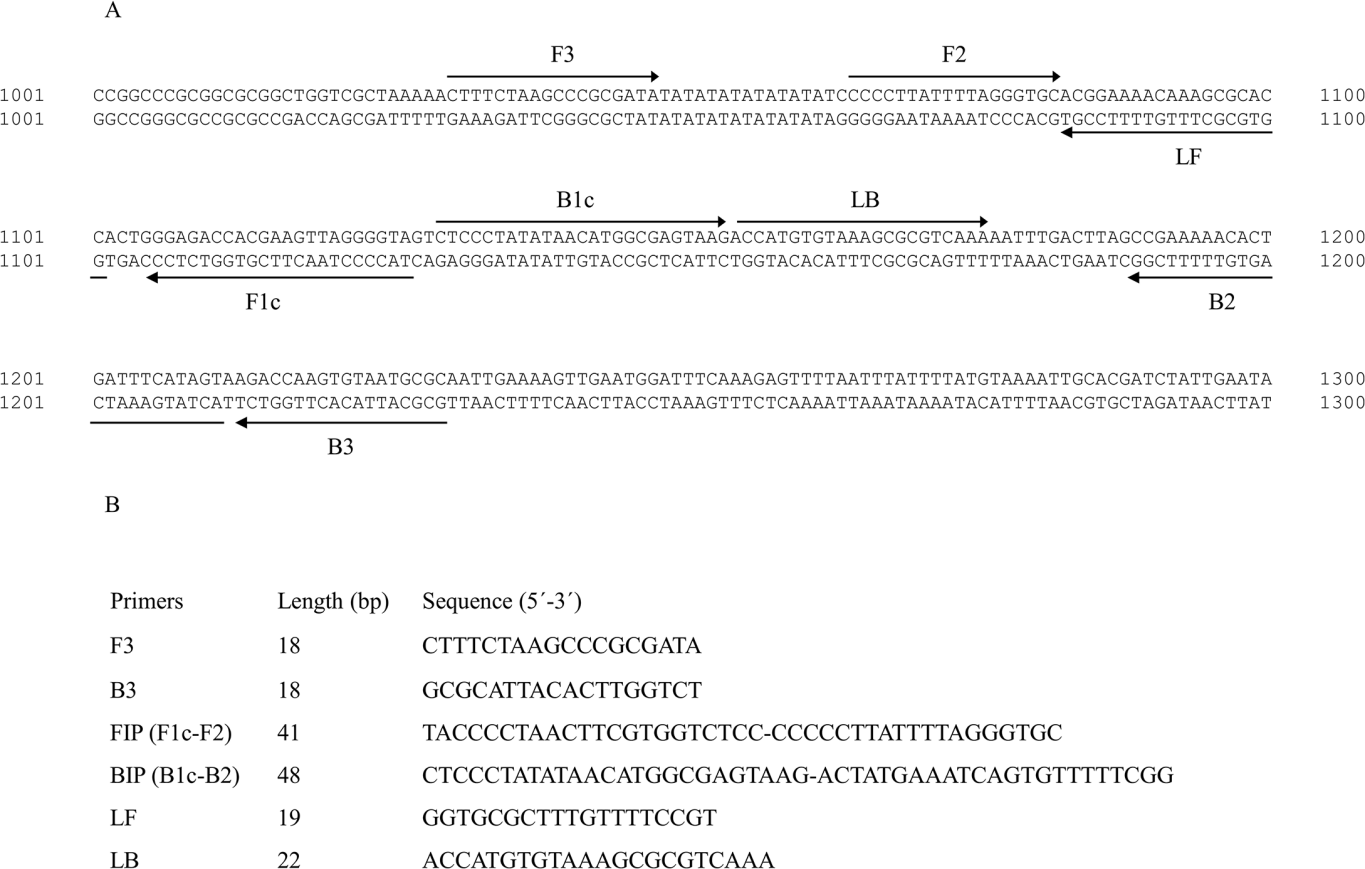
Lamp primer set targeting the selected sequence (GenBank Accession No. AJ223838) for ribosomal intergenic spacer *S*. *haematobium* DNA region amplification. (A) The location of the LAMP primers within the selected sequence is shown. Arrows indicate the direction of extension. (B). Sequence of LAMP primers: F3, forward outer primer; B3, reverse outer primer; FIP, forward inner primer (comprising F1c and F2 sequences); BIP, reverse inner primer (comprising B1c and B2 sequences); LF (loop forward primer); LB (loop backward primer).

A set of six oligonucleotide primers were used for the LAMP assay, targeting eight regions in the 2522 base pair (bp) sequence of *S*. *haematobium* ribosomal intergenic spacer (IGS) DNA retrieved from GenBank (Accession No. AJ223838) [55]. The outer forward primer (F3), outer backward primer (B3), forward inner primer (FIP), backward inner primer (BIP), and loop forward (LF) and backward (LB) primers were designed using the online Primer Explorer V4 software (Eiken Chemical Co. Ltd, Tokyo, Japan; http://primerexplorer.jp/elamp4.0.0/index.html) according to the general criteria described by Notomi et al. [32] and finally selected based on the criteria described in “A Guide to LAMP primer designing” (http://primerexplorer.jp/e/v4_manual/index.html). The location and nucleotide sequences of the six primers are shown in Fig 1. All the primers were of HPLC grade (Thermo Fisher Scientific Inc., Madrid, Spain). To confirm the specificity for the designed primers in annealing exclusively with the *S*. *haematobium* DNA correct target sequence, a BLASTN local search and alignment analysis [56] was carried out in different online databases against currently available nucleotide sequences for other organisms (NCBI; http://blast.ncbi.nlm.nih.gov/Blast.cgi) as well as specifically against human, murine (Ensembl; http://www.ensembl.org/Multi/Tools/Blast) and other related *Schistosoma* species genomes (Sanger Institute; http://www.sanger.ac.uk/resources/software/blast/).

### PCR using outer primers F3 and B3

The outer LAMP primer pair, designated F3 and B3, was initially tested for the amplification of *S*. *haematobium* DNA by a touchdown-PCR (TD-PCR) to verify whether the correct target was amplified. The PCR assay was conducted in 25 μL reaction mixture containing 2.5 μL of 10x buffer, 1.5 μL of 25 mmol/L MgCl_2_, 2.5 μL of 2.5 mmol/L dNTPs, 0.5 μL of 100 pmol/L F3 and B3, 2 U *Taq*-polymerase and 2 μL (1 ng) of DNA template. Conditions for TD-PCR amplification were as follows: an initial denaturation was conducted at 94°C for 1 min, followed by a touchdown program for 15 cycles with successive annealing temperature decrements of 1.0°C every 2 cycles. For these 2 cycles, the reaction was denatured at 94°C for 20 s followed by annealing at 58°C-55°C for 20 s and polymerization at 72°C for 30 s. The following 15 cycles of amplification were similar, except that the annealing temperature was 54°C. A final extension was performed at 72°C for 10 min.

The specificity of PCR using outer primers F3 and B3 was also tested with 20 heterogeneous DNA samples from other parasites included in the study. The sensitivity of the PCR was also assayed to establish the detection limit of *S*. *haematobium* DNA with 10-fold serial dilutions ranging from 0.5 ng/μL to 0.5 atg/μL prepared as mentioned above. The assays were performed with 2 μL of the diluted template in each case, thus resulting a final concentration of DNA ranging from 1 ng/μL to 1 atg/μL. Negative controls (ultrapure water instead of DNA template) were included in each run. The PCR products (5–10 μL) were subjected to 2% agarose gel electrophoresis stained with ethidium bromide and visualized under UV light.

### LAMP reaction

To evaluate the LAMP primer set designed in *S*. *haematobium* DNA amplification, we set up the reaction mixture using *Bst* 2.0 WarmStart DNA polymerase (New England Biolabs, UK) combined with different betaine (Sigma, USA) and MgSO_4_ (New England Biolabs, UK) concentrations. Thus, LAMP reactions mixtures (25 μL) contained 1.6 μM of each FIP and BIP primers, 0.2 μM of each F3 and B3 primers, 0.4 μM of each LB and LF primers, 1.4 mM of each dNTP (Bioron), 1x Isothermal Amplification Buffer -20 mM Tris-HCl (pH 8.8), 50 mM KCl, 10 mM (NH_4_)_2_SO_4_, 2 mM MgSO_4_, 0.1% Tween20- (New England Biolabs, UK), betaine (ranging 0.8, 1 or 1.2 M), supplementary MgSO_4_ (ranging 4, 6 or 8 mM) and 8 U of *Bst* 2.0 WarmStart DNA polymerase with 2 μL of template DNA. To establish the standard protocol for LAMP reactions mixtures assayed, a range of temperatures (61, 63 and 65°C) was tested in a heating block for 30, 50 or 60 min and then heated at 80°C for 5–10 min to inactivate the enzyme and thus to terminate the reaction. Then, both optimal temperature and incubation time were determined and used in the following tests. Positive (*S*. *haematobium* DNA) and negative (no DNA template) controls were always included in each LAMP assay.

#### Analysis of LAMP products

Firstly, when possible, turbidity caused by the accumulation of magnesium pyrophosphate (a by-product of the reaction) was visually inspected by the naked eyes. The positive amplification results were also visually detected by adding 2 μL of 1:10 diluted 10,000x concentration fluorescent dye SYBR Green I (Invitrogen) to the reaction tubes. Green fluorescence was clearly observed in a successful LAMP reaction, whereas it remained original orange in the negative one. After the LAMP reactions, 3–5 μL of each product were used for 2% agarose gel electrophoresis stained with ethidium bromide. A GelDoc imaging system (UVItec, UK) was used to observe the band patterns. The samples were considered positive if they showed a characteristic ladder-like band pattern.

#### Evaluation of LAMP assay

To evaluate the LAMP assay, we used the serially diluted artificial samples and also the patients´ urine samples with parasitological confirmed *S*. *haematobium* infection. The patients´ urine samples were tested by LAMP after being processed in different ways (as whole urine, supernatant and pellet) to test the most successful method for DNA extraction to be used as template as previously described. Once the most favorable urine fraction and DNA extraction method were established, LAMP assay was used to test twice all the human urine samples included in our study.

#### Specificity and sensitivity of the LAMP assay

The specificity of the LAMP assay to amplify only *S*. *haematobium* DNA was tested against a panel of 20 DNA samples obtained from other parasites used as controls, as mentioned above. On the other hand, in order to determine the lower detection limit of the LAMP assay, genomic DNA from *S*. *haematobium* 10-fold serially diluted-ranging from 0.5 ng/μL to 0.5 atg/μL, as mentioned above- was subjected to amplification. Moreover, the sensitivity was also assayed with the simulated urine samples artificially spiked with the same dilutions after DNA extraction by using the i-genomic Urine DNA Extraction Mini Kit (Intron Biotechnology, UK) and the Rapid-Heat LAMP method.

### Statistical analysis

To estimate the accuracy of the LAMP assay as a diagnostic test, the percentages of sensitivity, specificity, positive predictive value (PPV) and negative predictive value (NPV) were calculated using the MedCalc statistical program version 15.2.2 (MedCalc Software, Ostende, Belgium) according to the software instruction manual (www.medcalc.org).

## Results

### Sensitivity and specificity of PCR using outer primers

**Fig 2:**
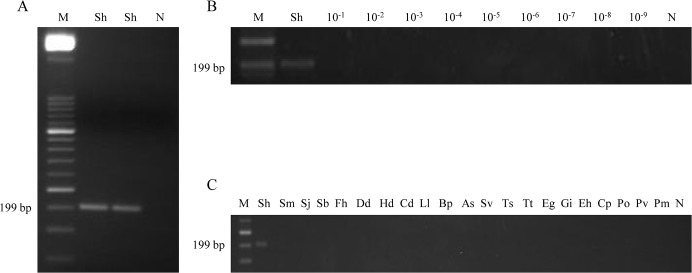
PCR verification, detection limit and specificity using outer primers F3 and B3. (A) PCR verification of expected 199 bp target length amplicon. Lane M, 50 bp DNA ladder (Molecular weight marker XIII, Roche); lane Sh, *S*. *haematobium* DNA (1 ng); lane N, negative control (no DNA template). (B) Detection limit of PCR. Lane M, 50 bp DNA ladder (Molecular weight marker XIII, Roche); lane Sh: *S*. *haematobium* DNA (1 ng); lanes 10^−1^–10^−9^: 10-fold serially dilutions of *S*. *haematobium* DNA; lane N, negative control (no DNA template). (C) Specificity of PCR. Lane M, 50 bp DNA ladder (Molecular weight marker XIII, Roche); lanes Sh, Sm, Sj, Sb, Fh, Dd, Hd, Cd, Ll, Bp, As, Sv, Ts, Tt, Eg, Gi, Eh, Cp, Po, Pv, Pm, *S*. *haematobium*, *S*. *mansoni*, *S*. *japonicum*, *S*. *bovis*, *Fasciola hepatica*, *Dicrocoelium dendriticum*, *Hymenolepis diminuta*, *Calicophoron daubneyi*, *Loa loa*, *Brugia pahangi*, *Anisakis simplex*, *Strongyloides venezuelensis*, *Trichinella spiralis*, *Taenia taeniformis*, *Echinococcus granulosus*, *Giardia intestinalis*, *Entamoeba histolytica*, *Cryptosporidium parvum*, *Plasmodium ovale*, *P*. *vivax* and *P*. *malariae* DNA samples (1 ng/each), respectively; lane N, negative control (no DNA template).

To confirm that the expected target was amplified, a PCR reaction was performed using outer primers F3 and B3 to amplify *S*. *haematobium* DNA. Thus, a 199 bp amplicon was successful obtained (Fig 2A). In order to determine the lower detection limit of the PCR reaction, a 10-fold serial dilution ranging from 10^−1^ to 10^−9^ of *S*. *haematobium* DNA was amplified. The minimum amount of DNA detectable by PCR using outer primers was 1 ng (Fig 2B). According to specificity, when DNA samples obtained from other parasites included in the study were subjected to this PCR assay, amplicons were never amplified (Fig 2C).

### Setting up LAMP assay

**Fig 3:**
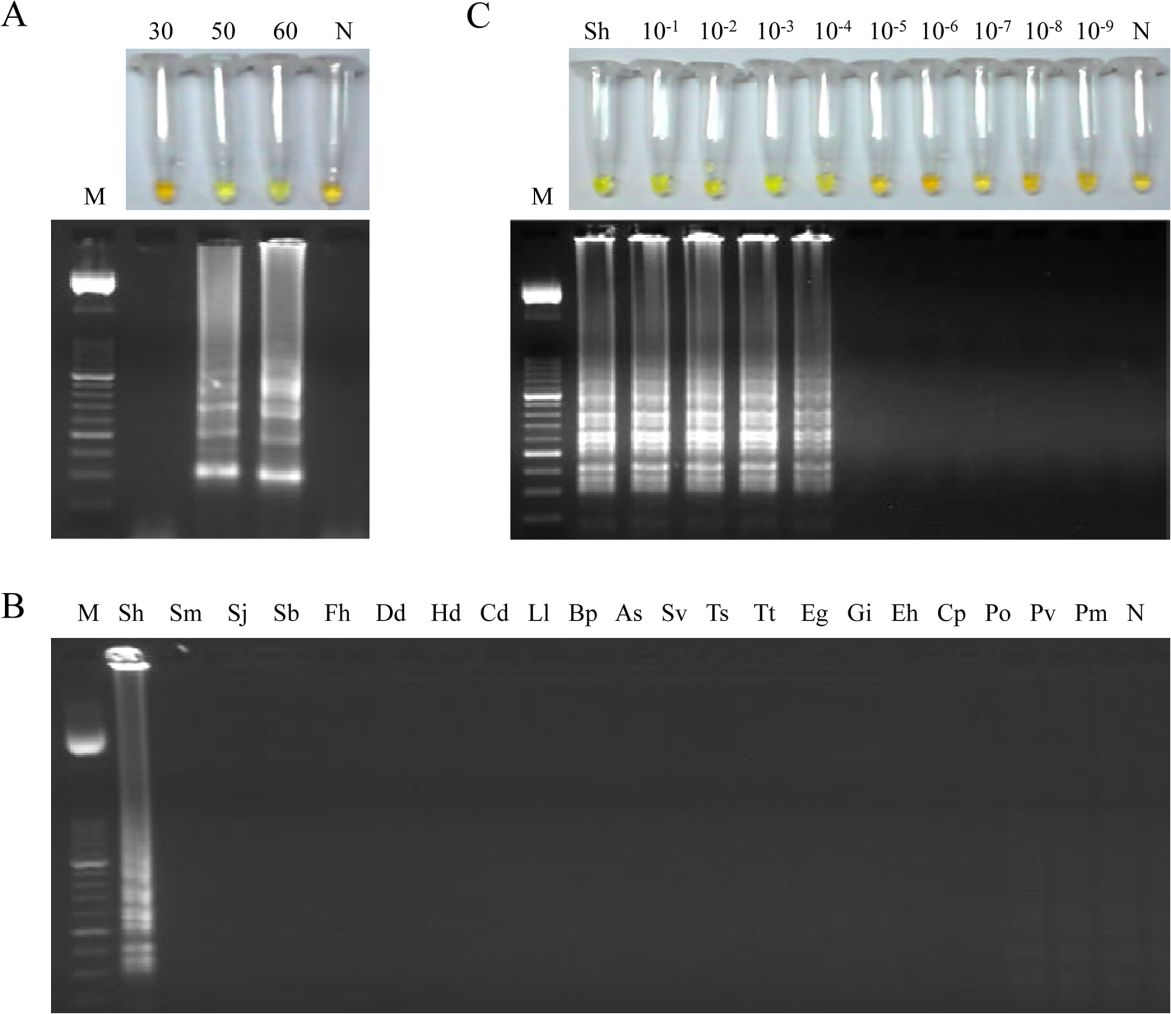
Setting up LAMP assay. (A) LAMP amplification results obtained at different incubation times (30, 50 and 60 min) tested in a heating block by the addition of SYBR Green I (up) or by visualization on agarose gel (down). Lane M, 50 bp DNA ladder (Molecular weight marker XIII, Roche); lanes 30, 50, 60, amplification results of *S*. *haematobium* DNA (1 ng) for 30, 50 and 60 minutes of incubation time, respectively. (B) Specificity of the LAMP assay for *S*. *haematobium*. A ladder of multiple bands of different sizes could be only observed in *S*. *haematobium* DNA sample. Lane M, 50 bp DNA ladder (Molecular weight marker XIII, Roche); lanes Sh, Sm, Sj, Sb, Fh, Dd, Hd, Cd, Ll, Bp, As, Sv, Ts, Tt, Eg, Gi, Eh, Cp, Po, Pv and Pm, *S*. *haematobium*, *S*. *mansoni*, *S*. *japonicum*, *S*. *bovis*, *Fasciola hepatica*, *Dicrocoelium dendriticum*, *Hymenolepis diminuta*, *Calicophoron daubneyi*, *Loa loa*, *Brugia pahangi*, *Anisakis simplex*, *Strongyloides venezuelensis*, *Trichinella spiralis*, *Taenia taeniformis*, *Echinococcus granulosus*, *Giardia intestinalis*, *Entamoeba histolytica*, *Cryptosporidium parvum*, *Plasmodium ovale*, *P*. *vivax* and *P*. *malariae* DNA samples (1 ng/each), respectively; lane N, negative control (no DNA template). (C) Sensitivity assessment performed with LAMP at 63°C for 50 min using serial dilutions of *S*. *haematobium* genomic DNA. Lane M: 50 bp DNA ladder (Molecular weight marker XIII, Roche); lanes Sh: genomic DNA from *S*. *haematobium* (1 ng); lanes 10^−1^–10^−9^: 10-fold serially dilutions; lane N: negative controls (no DNA template).

To establish a standard procedure for the LAMP assay we used the *Bst* 2.0 WarmStart DNA polymerase applying a range of temperatures (61, 63 and 65°C) for testing different mixtures containing variable concentrations of betaine (ranging 0.8, 1 or 1.2 M) combined with supplementary variable concentrations of MgSO4 (ranging 4, 6 or 8 mM) in a heating block for 30, 50 and 60 min. The best amplification results were obtained when the reaction mixture contained 1 M of betaine combined with supplementary 6 mM of MgSO4 (resulting a final concentration of 8 mM MgSO4 in 1x Isothermal Amplification Buffer) and was incubated for 50 min at 63°C in a heating block (Fig 3A). Once the most favourable conditions and molecular components were established for the LAMP assay, all positive results in subsequent reactions could be clearly visually observed by the naked eye by inspecting the colour change after adding SYBR Green I as well as the typical ladder of multiple bands after electrophoresis on agarose gels.

### Specificity and sensitivity of LAMP assay

To determine the specificity of the primers designed, a panel of 20 DNA samples from other parasites were subjected to the LAMP assay. As shown in Fig 3B, only LAMP products were amplified when *S*. *haematobium* DNA was used as template and no false positive amplification was observed, thus indicating the high specificity of the established LAMP assay.

**Fig 4:**
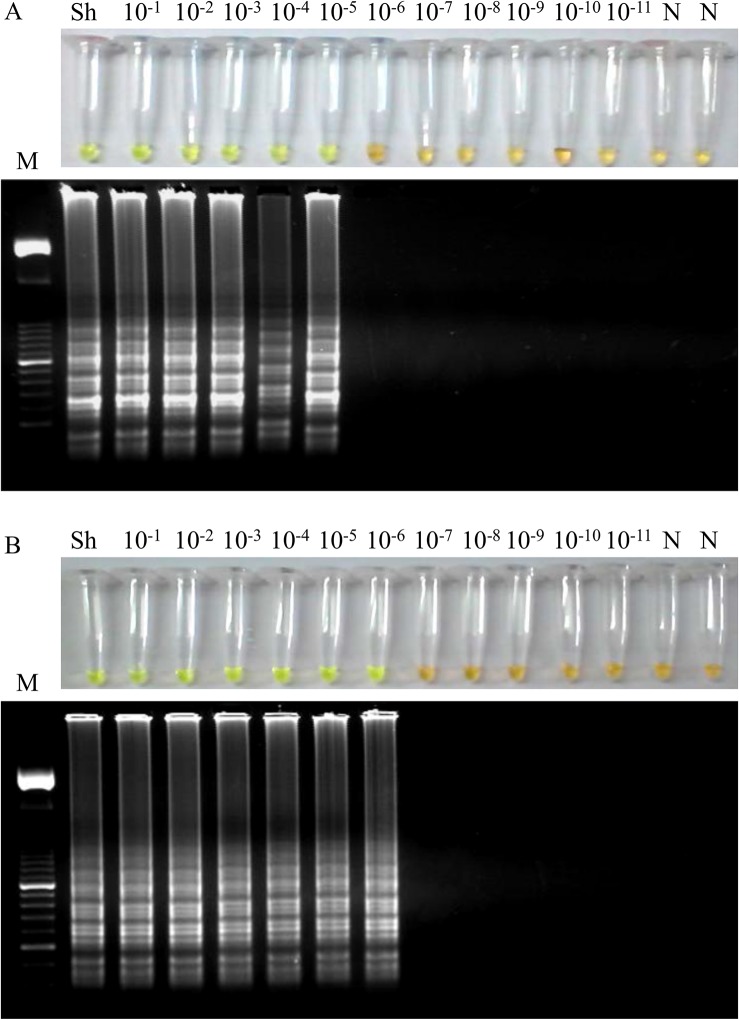
Sensitivity of the LAMP assay in simulated human urine samples artificially contaminated with DNA from *S*. *haematobium*. (A) Sensitivity assessment of LAMP when performing the DNA extraction with the i-genomic Urine DNA Extraction Mini Kit (Intron Biotechnology, UK) from serial dilutions of *S*. *haematobium* genomic DNA. (B) Sensitivity assessment of LAMP when performing the DNA extraction with a simple heating method from serial dilutions of *S*. *haematobium* genomic DNA. Lanes M: 50 bp DNA ladder (Molecular weight marker XIII, Roche); lanes Sh: genomic DNA from *S*. *haematobium* (1 ng); lanes 10^−1^–10^−11^: 10-fold serially dilutions; lanes N: negative controls (no DNA template).

Regarding to the sensitivity of the LAMP assay, a 10-fold serial dilution of *S*. *haematobium* genomic DNA was amplified by LAMP. The results indicated that the detection limit for the LAMP reaction was 100 fg (Fig 3C). This suggested that the LAMP assay is 10^4^ times more sensitive than the PCR using outer primers F3 and B3 (see Fig 2B). On the other hand, the sensitivity of LAMP assay in simulated fresh human urine samples artificially contaminated with DNA from *S*. *haematobium* was also examined. In this case, the detection limit of LAMP assay was 10 fg/μL when performing the DNA extraction with the commercial kit (Fig 4A), whereas the detection limit was established in 1 fg/μL using the Rapid-Heat LAMPellet method for DNA extraction (Fig 4B).

### LAMP tests in confirmed *S*. *haematobium-*positive patients´ urine samples

**Fig 5:**
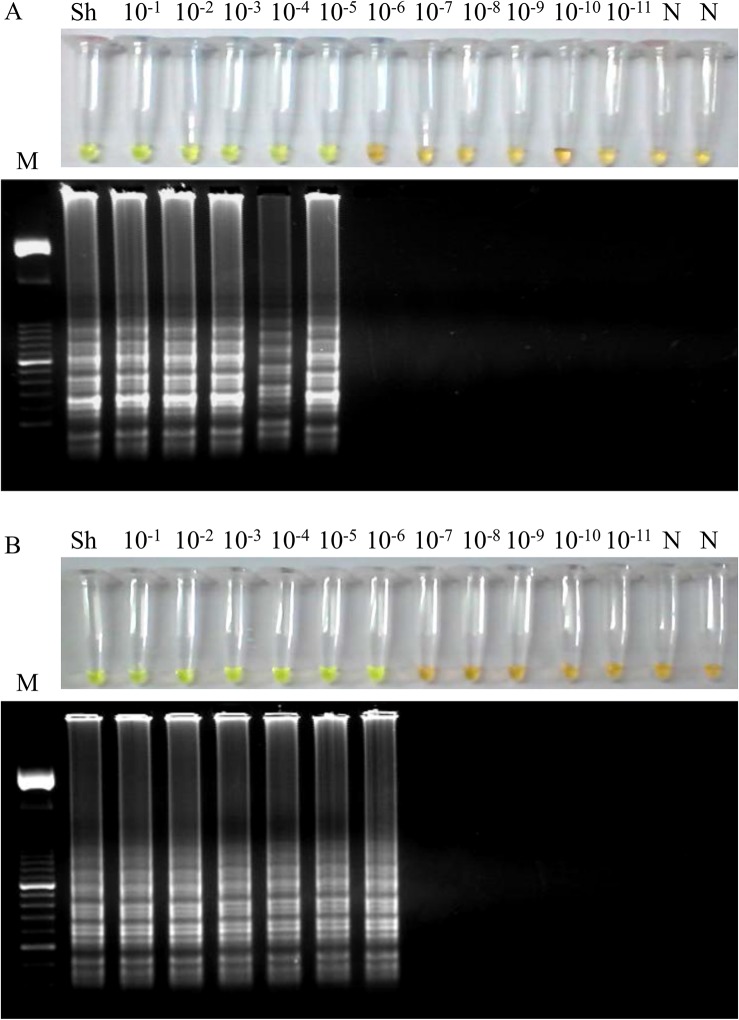
Examination of aliquots of whole urine from *S*. *haematobium*-positive patients´ urine samples by LAMP. Figure shows the LAMP results (up, by color change; down, by agarose electrophoresis) when using aliquots of 100 μL of whole urine to obtain DNA as template by using (A) the i-genomic Urine DNA Extraction Mini Kit (Intron Biotechnology, UK); (B) the heating NaOH-SDS method and (C) the rapid heating method. Lanes M: 50 bp DNA ladder (Molecular weight marker XIII, Roche); lanes Sh: genomic DNA from *S*. *haematobium* (1 ng); lanes 1–18: *S*. *haematobium*-positive samples; lanes N: negative controls (no DNA template).

**Fig 6:**
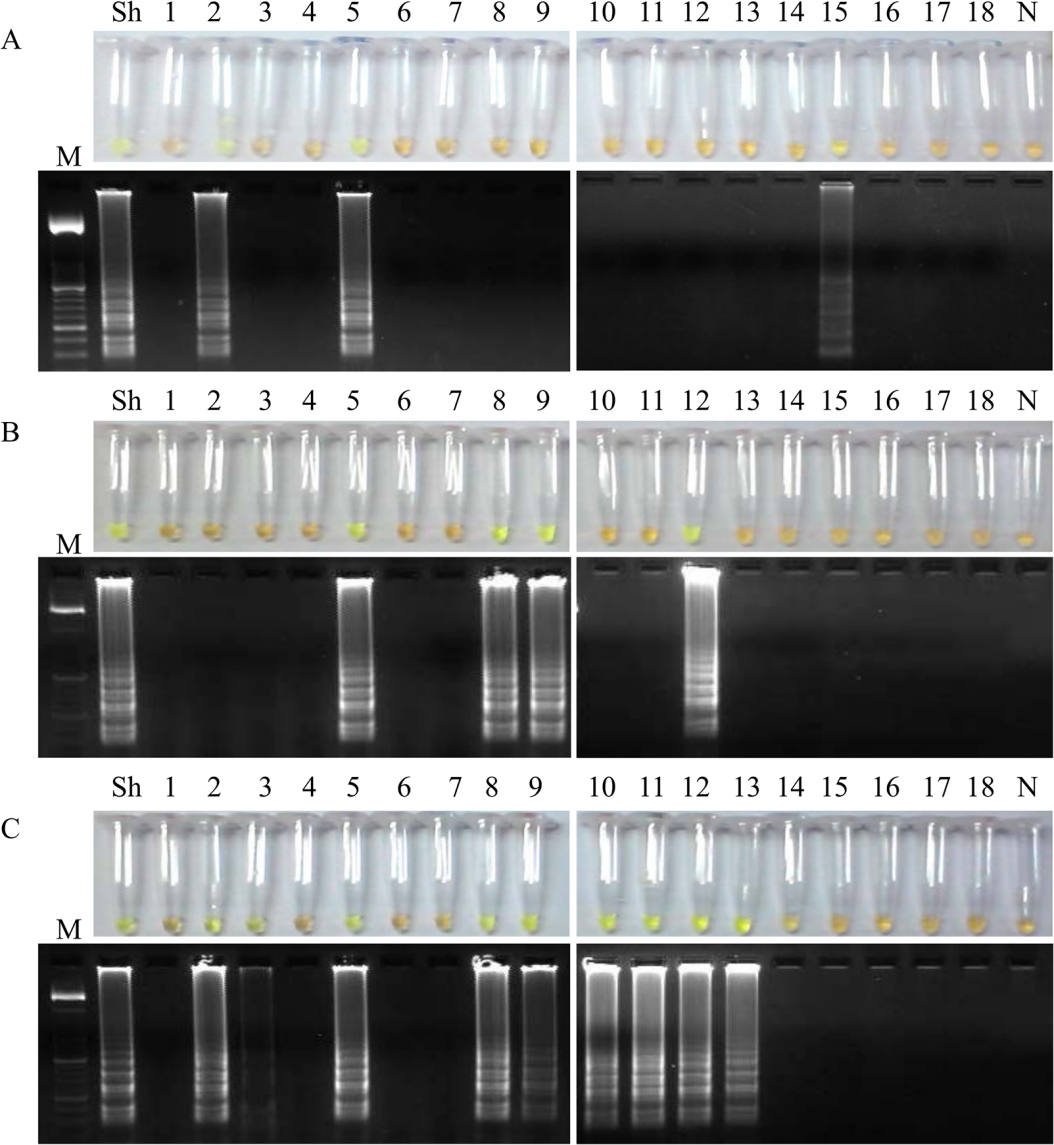
Examination of aliquots of supernatants from *S*. *haematobium*-positive patients´ urine samples by LAMP. Figure shows the LAMP results (up, by color change; down, by agarose electrophoresis) when using aliquots of 100 μL of supernatants to obtain DNA as template by using (A) the i-genomic Urine DNA Extraction Mini Kit (Intron Biotechnology, UK); (B) the heating NaOH-SDS method and (C) the rapid heating method. Lanes M: 50 bp DNA ladder (Molecular weight marker XIII, Roche); lanes Sh: genomic DNA from *S*. *haematobium* (1 ng); lanes 1–18: *S*. *haematobium*-positive samples; lanes N: negative controls (no DNA template).

**Fig 7:**
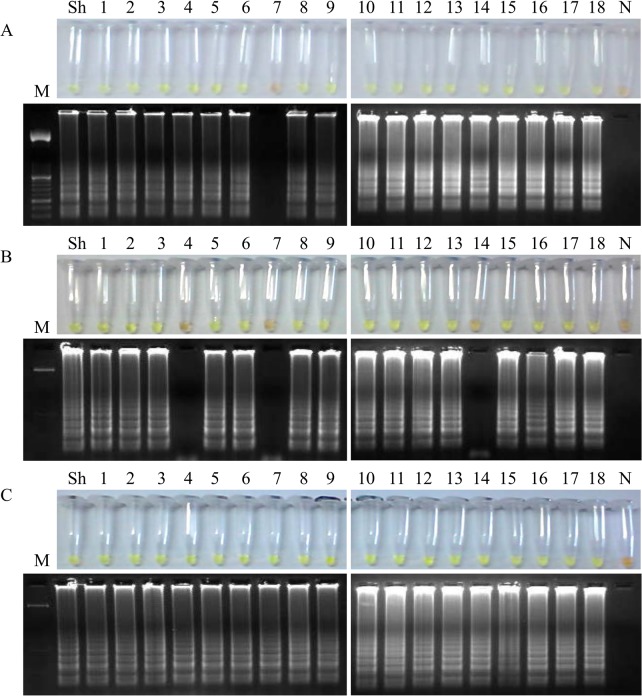
Examination of aliquots of urinary sediment (pellets) from *S*. *haematobium*-positive patients´ urine samples by LAMP. Figure shows the LAMP results (up, by color change; down, by agarose electrophoresis) when using aliquots of 100 μL of pellets to obtain DNA as template by using (A) the i-genomic Urine DNA Extraction Mini Kit (Intron Biotechnology, UK); (B) the heating NaOH-SDS method and (C) the rapid heating method-the rapid-heat LAMPellet method-. Lanes M: 50 bp DNA ladder (Molecular weight marker XIII, Roche); lanes Sh: genomic DNA from *S*. *haematobium* (1 ng); lanes 1–18: *S*. *haematobium*-positive samples; lanes N: negative controls (no DNA template).

Comparative LAMP results obtained when testing aliquots of whole urine, supernatants and pellets from patients´ urine samples with parasitological confirmed *S*. *haematobium* infection after using the three different DNA extraction methods attempted in our study are shown in Figs 5, 6 and 7, respectively.

In LAMP tests using a starting volume of whole patients´ urine samples of 100 μL/each we obtained 15/18 positive results when performing DNA extraction using the i-genomic Urine DNA Extraction Mini Kit (Fig 5A), 11/18 when using the NaOH/SDS extraction method (Fig 5B) and 12/18 when the Rapid-Heat LAMP method was applied (Fig 5C).

In LAMP tests for supernatant fraction of patients´ urine samples we obtained only 3/18 positive results when performing DNA extraction using the i-genomic Urine DNA Extraction Mini Kit (Fig 6A), 4/18 when using the NaOH/SDS extraction method (Fig 6B) and 9/18 when the Rapid-Heat LAMP method was applied (Fig 6C).

Finally, in LAMP tests for the urinary sediment (pellet) obtained from the urine samples we obtained 17/18 positive results when performing DNA extraction using the i-genomic Urine DNA Extraction Mini Kit (Fig 7A), 15/18 when using the NaOH/SDS extraction method (Fig 7B) and a total of 18/18 when the Rapid-Heat LAMP method was applied (Fig 7C). Thus, in general, the higher effectiveness in LAMP amplification of *S*. *haematobium* DNA in patients´ urine samples was obtained when the urinary sediment (pellet) was used for DNA extraction; moreover, the simple Rapid-Heat LAMP method provided the best results of the three methods assayed for extracting DNA detectable by LAMP. Thereby, the minimal pellet obtained from urine samples, in addition to the Rapid-Heat LAMP method for DNA detection-hereafter "Rapid-Heat LAMPellet method"-, was set up as the most advantageous procedure to be used in successive LAMP reactions to detect *S*. *haematobium* DNA in urine samples and to test all the clinical samples included in our study.

### Rapid-Heat LAMPellet method in clinical sample collection

The results of all 94 patients´ urine samples evaluated by duplicated for *S*. *haematobium* DNA detection by using the Rapid-Heat LAMPellet method are presented in Table 1. We obtained LAMP positive results in 18/18 confirmed *S*. *haematobium* infected urine samples, in 1/9 urine samples with other helminths species confirmed infections (specifically a patient infected with a "hookworm"), in 1/5 urine samples with other agents confirmed infections (specifically a patient infected with *Trichomonas vaginalis*), in 1/15 urine samples from patients with eosinophilia without a confirmed diagnosis and, finally, in 5/24 urine samples from patients without either eosinophilia and none apparent disease. The seven parasitological *S*. *mansoni*-positive urine samples as well as the 16 urine samples from healthy non-endemic donors (used as negative controls samples) were all negative by LAMP. All positive results could be visually observed in tubes by color change after adding SYBR Green I and also after electrophoresis on agarose gels as a ladder of multiple bands of different sizes (S1 Fig). Considering the results obtained, diagnostic parameters for sensitivity and specificity were calculated for our LAMP assay, 100% sensitivity and 86.67% specificity, and also for microscopy detection of eggs in urine samples, 69.23% sensitivity and 100% specificity. The PPV and NPV for both LAMP assay and microscopy were also calculated; all statistic data obtained are showed in Table 1.

**Table 1:** Estimation of sensitivity, specificity, predictive values and likelihood ratios by Rapid-Heat LAMPellet method against standard parasitological test (microscopy) for current study for identifying *Schistosoma haematobium* infection in patients´ urine samples.

Diagnostic test	Sensitivity (95% CI)	Specificity (95% CI)	PPV (95% CI)	NPV (95% CI)
Rapid-Heat LAMPellet	100% (81.32%-100%)	86.67% (75.40%-94.05%)	63.23% (48.21%-85.63%)	100% (93.08%-100%)
Microscopy	69.23% (48.21%-85.63%)	100% (93.08%-100%)	100% (81.32%-100%)	86.67% (75.40%-94.05%)

PPV, Positive Predictive Value

NPV, Negative Predictive Value

## Discussion

Urogenital schistosomiasis due to S. *haematobium* remains a serious underestimated public health problem, particularly in sub-Saharan Africa. Frequency of urogenital schistosomiasis in travellers, expatriates and migrants is in the same range to that of intestinal schistosomiasis due to *S*. *mansoni* [57]. As there is no vaccine to protect against schistosomal infection, mass praziquantel treatment of populations at risk of infection is being conducted routinely in many endemic areas; however, reinfections rapidly occur because of recurrent direct contact with water infected with parasites [58]. Considering the current problems of parasitological, serological and molecular methods in detecting schistosomal infections [59], new, simple, accurate and affordable diagnostic tools are essential for providing specific treatment and for maximizing the success of control of urogenital schistosomiasis in endemic areas; as well as for monitoring drug effectiveness.

Point-of-care tests are being developed as economic evaluation diagnostic technologies for infectious diseases control strategies as they are easy to use and interpret, require minimal laboratory infrastructure, are cost-effective, reduce patient waiting time and potentially therefore reduce loss to follow-up, and may have comparable or higher sensitivity to microscopy [60]. The LAMP technology-as a DNA amplification method- combines rapidity, simplicity and high specificity [32] and has a wide range of possible applications, including point-of-care testing in developing countries [61, 62]. We have developed a LAMP assay for rapid, sensitive, specific and cost-effective detection of *S*. *haematobium* in human urine samples, even in the absence of parasites eggs in excreta, as a basis for a potential field diagnostic tool for use in schistosomiasis endemic areas. Besides its excellent performance, the most striking results of this study are the simplicity to perform the whole process without requiring DNA extraction from a small volume of starting urine to get the urinary sediment (pellet) to carry out the molecular analysis. We have named this simple procedure the "Rapid-Heat LAMPellet method".

To accomplish its development, we designed a specific set of six primers targeting eight regions in a species specific sequence of *S*. *haematobium* ribosomal IGS [55]. The ribosomal IGS regions within *Schistosoma* species generally contain unique sequence motifs which are specific to that group of organisms. In addition, the IGS target locus has been already used for successful detection of *Schistosoma* spp. infection in freshwater snails by real-time PCR and oligochromatographic dipstick rapid technology (PCR-OC) [63]. Several other advantages of these sequences to be use in molecular studies have been already reported elsewhere [55, 64].

Once the primer set was designed, *in silico* comparisons of the expected 199 bp sequence with the on line available genomes showed the higher homology in alignment length with *S*. *haematobium* and no cross-reaction was found, specifically with *S*. *mansoni*; this result is especially important as these two species are the main schistosomes producing co-infections in most areas of sub-Saharan Africa [58]. Specificity results obtained in *in silico* were later verified by PCR using outer primers F3-B3.

After this, we attempted to establish the most suitable reaction mixture for the six specific primers in the LAMP assay. We used the *Bst* polymerase 2.0 WarmStart as this warm-start version has several advantages compared to wild-type *Bst* DNA polymerase large fragment, such as faster in obtaining amplification signals [65] and increased stability at room temperature [66]. These features are important when testing a large number of samples under field conditions in endemic areas where limited resources for the maintenance of a cold chain exists. As the LAMP reaction might be facilitated by the addition of loop primers [67] our LAMP assay designed was accelerated by the addition of a pair of loop primers, thus allowing to amplify successfully *S*. *haematobium* DNA in only 50 min, whereas a previously described LAMP assay to amplify *S*. *haematobium* DNA in freshwater snails takes 120 min to complete the reaction [49, 50].

The specificity of the LAMP assay was determined using a panel of heterogeneous control DNA samples of a number of parasites. The assay specifically produced typical ladder patterns from the target sequence only for *S*. *haematobium* DNA. The sensitivity of the LAMP resulted 10^4^ times greater than that of PCR using outer primers (100 fg *vs*. 10^6^ fg or 1ng, respectively). It is usually considered that LAMP is highly sensitive compared to conventional PCR methods and other studies also found a higher sensitivity when comparing LAMP results in contrast to PCR in amplification of DNA from *Schistosoma* species, including *S*. *japonicum* [47], *S*. *haematobium* and *S*. *mansoni* [49, 48].

The effectiveness of our LAMP assay was assessed in patients´ urine samples with confirmed *S*. *haematobium* infection by microscopic examination. Bearing in mind a potential easy and cost-effective large-scale application in field conditions, we evaluated different DNA extraction methods for their ability to isolate DNA from small volumes of different fractions of human urine samples, including whole urine, urine supernatant and urinary sediment (pellet) to compare results. A simple, quick and economically DNA extraction method for use in combination with small volumes of clinical urine specimens could greatly reduce the infrastructure requirements of collecting, handling, storing and processing the patients´ samples in schistosomiasis endemic areas where limited resources exist.

The three different DNA extraction methods tested in our work were much more efficient in extracting detectable DNA by LAMP when using aliquots of whole urine and pellets than supernatants. This seems to be logical since after centrifugation to remove and retain supernatants, both potential free *S*. *haematobium* DNA and parasite eggs-and therefore containing DNA- found in patients´ urine samples should be concentrated at the bottom of the tube, thus improving the sensitivity of the DNA molecular detection methods, as previously described [68]. When using the pellets, the simple rapid-heating method allowed us to obtain a very good-quality detectable DNA that did not compromise LAMP amplification and all the *S*. *haematobium*-positive urine samples tested were successfully amplified.

The consistent results in DNA obtained from aliquots of whole urine and pellets when applying a commercial kit may be due to the well-known effectiveness of this procedure to isolate genomic DNA from urine samples suitable for further molecular analyses [69]. Urine specimens contain many inhibitors which may interfere in DNA amplification [70], so removing inhibitors as much as possible by using a kit is convenient to ensure that DNA will be subsequently efficiently amplified. However, since this procedure could be very expensive to use when a large number of samples must be tested, an inexpensive and simple rapid-heating method is much more advantageous. It is also known that DNA purification from samples could be omitted in LAMP reactions, since LAMP assays have shown a significant tolerance to inhibitor substances derived from a number of biological samples [71], [72], [73]. Additionally, other LAMP assays with high sensitivity and no complicate requirement procedure for DNA extraction have been developed for molecular detection and diagnostic of bacterial [74] and parasitic [75] diseases in urine samples. Moreover, a simple heating DNA obtaining method has been also successfully applied with other clinical samples, such us blood [41] and swaps [42] in LAMP amplification of both *Plasmodium* and *Leishmania* species nucleic acids, respectively.

To really establish the sensitivity of our LAMP assay in urine samples that most closely resembled the patients´ urine specimens analyzed, we used a panel of simulated human urine samples artificially spiked with *S*. *haematobium* genomic DNA. For these samples, to extract DNA as template in LAMP we used both the commercial kit and the rapid-heat methods since these procedures showed the highest efficiency to obtain detectable DNA by LAMP in *S*. *haematobium*-positive clinical samples. After extracting DNA with the commercial kit, LAMP detection limit resulted tenfold higher than that obtained using *S*. *haematobium* genomic DNA 10-fold serially diluted without DNA extraction (10 fg *vs*. 100 fg, respectively). Unexpectedly, when heating the simulated samples, we obtained a limit of detection tenfold higher than that obtained when using purified DNA samples by the commercial kit (corresponding to 1 fg *vs*. 10 fg, respectively). An increased sensitivity has been also reported when using crude DNA extraction methods compared with a commercial method (i.e. DNazol) for template preparation from the pellets or supernatants of nasopharyngeal aspirates for LAMP detection of adenovirus [76]. Thus, the sensitivity value of 1 fg was considered as the lower limit of the detection threshold of the LAMP assay in detecting *S*. *haematobium* DNA in human urine samples. By reference, as *S*. *mansoni* genome contains approximately 580 fg of DNA [77], theoretically our LAMP assay would detect *S*. *haematobium* diluted DNA in urine samples corresponding to less than the equivalent to a single parasite cell. Such sensitivity is a feature of great value to overcome the difficulties of detecting urogenital schistosomiasis in areas of low transmission or in individual cases with a very low worm burden.

Then, taking into account both the high sensitivity and the good-quality detectable *S*. *haematobium* DNA by LAMP in easy to obtain and handling heated pellets from clinical urine samples, we tested the remaining 76 specimens included in our study by the Rapid-Heat LAMPellet method. We obtained negative results by LAMP in all parasitologically *S*. *mansoni*-positive urine samples tested (corroborating again that no cross-reaction with that schistosome species occurs) and also in urine samples from healthy non-endemic donors used as negative controls. Nevertheless, eight LAMP positive results were obtained when testing patients´ urine samples from other groups which were formerly microscopy-confirmed as *S*. *haematobium*-negative. It may be rational to consider that those eight LAMP positive results are truly *S*. *haematobium*-infected samples which were undetected in the microscopic analysis since this method is very low sensitive, especially in low-grade infections and high day-to-day variable. Regarding the two LAMP positive results in patients´ urine samples with other microscopy-confirmed infectious diseases (i.e. hookworm and *T*. *vaginalis*), it is not uncommon to find co-infections of *S*. *haematobium* with other organisms such as bacteria, protozoa and helminths, including the hookworms [78]. It is unlikely that this result is due to a cross-reaction with hookworm since we obtained LAMP negative results in other three patients´ urine samples with microscopy-confirmed infection with this geohelminth. One eosinophilic without confirmed diagnosis patient as well as five non-eosinophilic without apparent pathologic disease individuals had *S*. *haematobium*-positive results by LAMP. The presence of absence of eosinophils is usually used as a biomarker for helminthic infections, including schistosomiasis [79]; however, it is not predictive of *Schistosoma* species infection and may generate inconsistent results [80]. Thus, application of our LAMP method may improve the identification of cases with low-intensity infections as well as in cases which did not pass eggs in urine samples, thus revealing infections in people frequently presumed to be uninfected. Finally, although all patients´ urine samples were tested in duplicate with the same result, it would be very interesting to know how reproducible the technique is when testing in field settings as well.

In conclusion, we have demonstrated that simply rapid-heating urinary pellets for good-quality DNA extraction was effective for use in LAMP assays with regard of detecting *S*. *haematobium* in clinical urine samples. This procedure has been named the Rapid-Heat LAMPellet method and it would be well-suited to diagnose urogenital schistosomiasis in resource-limited endemic regions because of its rapidity, easy handling, cost-effectiveness and both high detection specificity and sensitivity. The next step for refining the assay by conducting a field evaluation in an endemic setting should be desirable.

## Supporting Information

S1 Checklist STARD checklist. (PDF)

S1 Fig Examination of the patients´ urine samples by the Rapid-Heat LAMPellet method. Figure shows the LAMP results (up, by color change; down, by agarose electrophoresis) when testing clinical urine samples from different groups of patients included in our study by using heated pellet following by the specific LAMP assay for *S*. *haematobium* DNA detection. (A) Urine samples from patients with confirmed infection with several helminths. (B) Urine samples from patients with confirmed infection with different infectious agents (protozoa, bacteria and virus). (C) Urine samples from patients with eosinophilia but not confirmed diagnosis. (D) Urine samples from patients without either eosinophilia and none apparent disease. (E) Urine samples from patients with confirmed *S*. *mansoni* infection. (F) Urine samples from healthy non-endemic individuals (negative controls). Lanes M: 50 bp DNA ladder (Molecular weight marker XIII, Roche); lanes Sh: genomic DNA from *S*. *haematobium* (1 ng); lanes numbered, number of urine samples included in each group of patients.
(TIFF)

S1 Flowchart A diagram showing experimental design and results. (TIFF)
