# Supplementary figures and images for: The Rapid-Heat LAMPellet Method: A Potential Diagnostic Method for Human Urogenital Schistosomiasis
Source: PLoS Negl Trop Dis. 2015 Jul 31;9(7):e0003963. doi: 10.1371/journal.pntd.0003963 (PMC4521856; doi:10.1371/journal.pntd.0003963)

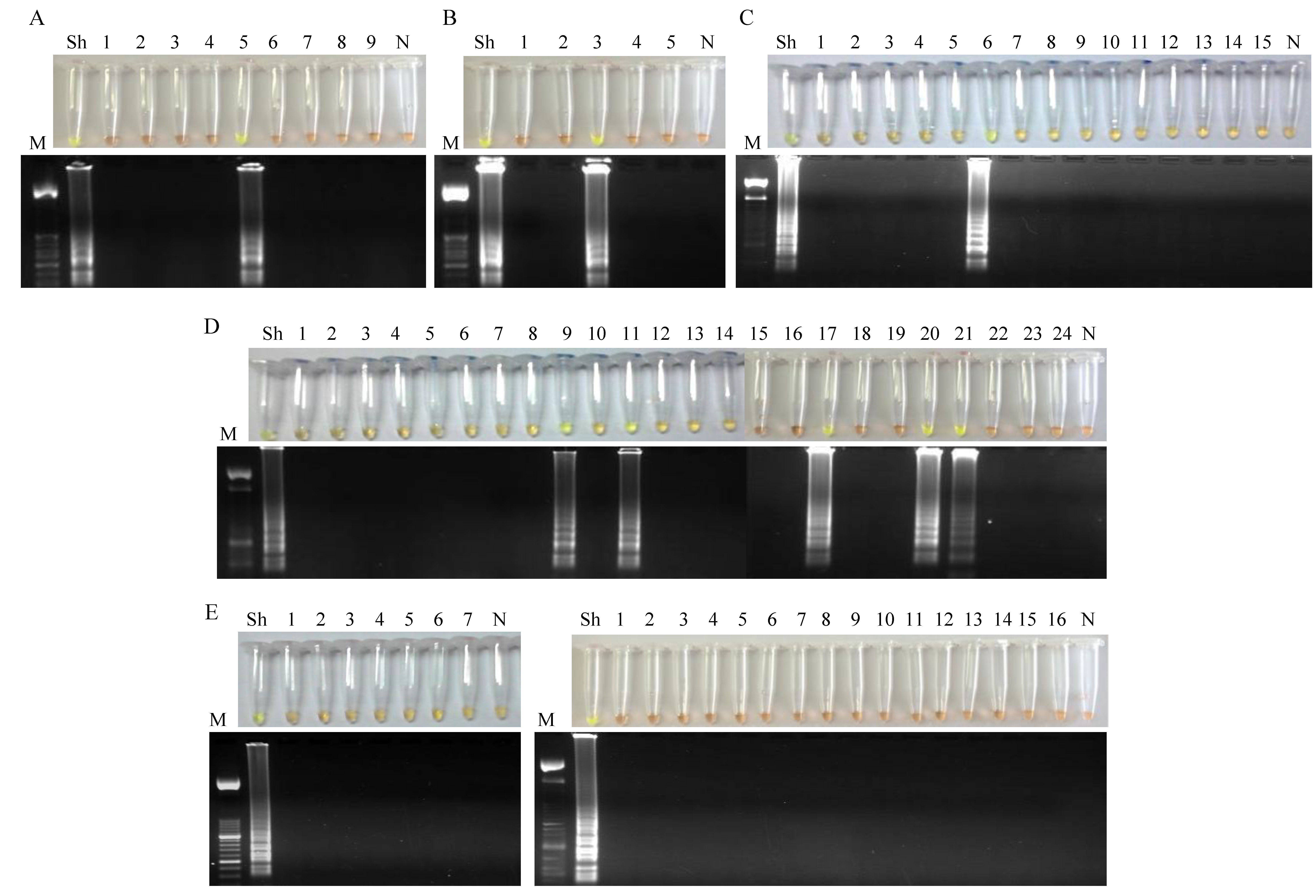

Supplement: S1 Fig — Figure shows the LAMP results (up, by color change; down, by agarose electrophoresis) when testing clinical urine samples from different groups of patients included in our study by using heated pellet following by the specific LAMP assay for S. haematobium DNA detection. (A) Urine samples from patients with confirmed infection with several helminths. (B) Urine samples from patients with confirmed infection with different infectious agents (protozoa, bacteria and virus). (C) Urine samples from patients with eosinophilia but not confirmed diagnosis. (D) Urine samples from patients without either eosinophilia and none apparent disease. (E) Urine samples from patients with confirmed S. mansoni infection. (F) Urine samples from healthy non-endemic individuals (negative controls). Lanes M: 50 bp DNA ladder (Molecular weight marker XIII, Roche); lanes Sh: genomic DNA from S. haematobium (1 ng); lanes numbered, number of urine samples included in each group of patients. (TIFF) [file pntd.0003963.s002.tiff]
